# ZAG promotes colorectal cancer cell proliferation and epithelial–mesenchymal transition by promoting lipid synthesis

**DOI:** 10.1515/biol-2022-1007

**Published:** 2024-12-18

**Authors:** Maotao Xu, Xingzheng Jin, Zhouli Shen

**Affiliations:** Department of Gastroenterology, The Ninth People’s Hospital of Chongqing, Chognqing, 400700, China; Department of Surgery, Southwest University Hospital, Chongqing, 400700, China; Department of Gastroenterology, The Ninth People’s Hospital of Chongqing, No. 69, Jialing Village, Beibei District, Chognqing, 400700, China

**Keywords:** colorectal cancer, ZAG, epithelial-mesenchymal transition, lipid production, PI3K/AKT/mTOR

## Abstract

Colorectal cancer (CRC) is a common malignant tumor characterized by a high degree of invasiveness, and since zinc-α2 glycoprotein (ZAG) has been implicated in the progression of several malignancies, this study was designed to investigate the role of ZAG in CRC. Its expression was assessed using the GEPIA database, and short hairpin RNA (shRNA) interference was conducted to create ZAG knockdown in CRC cell lines. We also conducted lipid synthesis, cell proliferation, apoptosis, and epithelial–mesenchymal transition (EMT) experiments to elucidate the effects of ZAG expression on CRC, as well as explored the potential underlying mechanistic pathways. Our findings reveal that ZAG is overexpressed in CRC. *In vitro*, ZAG knockdown resulted in the suppression of lipid production, cell division, and EMT while concurrently promoting apoptosis. The phosphoinositide 3-kinase (PI3K)/protein kinase B (AKT)/mechanistic target of rapamycin (mTOR) signaling pathway was found to mediate the effects of ZAG on CRC cells. In conclusion, the downregulation of ZAG can inhibit CRC cell survival, EMT, and lipid production via the PI3K/AKT/mTOR signaling pathway.

## Graphical Abstract

**Figure:**
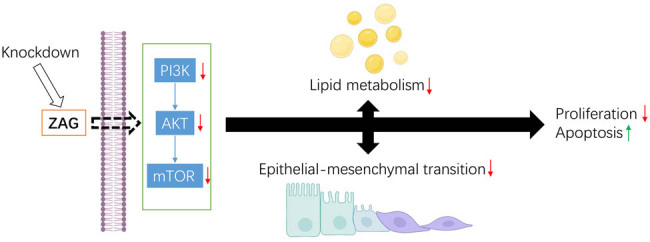

## 1 Introduction

Colorectal cancer (CRC) is one of the most prevalent cancers globally, accounting for approximately 8% of new cancer cases each year, with over 130,000 cases reported annually, ranking second in incidence among men and third among women [1,2]. The development of CRC is influenced by a combination of genetic, epigenetic, and environmental factors, and its pathogenesis involves anomalies in signaling pathways, lipid metabolic disorders, genetic alterations, and epigenetic modifications [3,4]. As various genetic and epigenetic changes, including histone modifications, DNA methylation, and non-coding RNAs, gradually accumulate in normal colonic epithelium, colorectal adenomas and invasive adenocarcinomas emerge. An increasing number of studies have highlighted the potential of targeting epigenetic alterations for therapeutic intervention, which may form the basis for individualized precision medicine strategies in the future [5,6]. In addition, dietary factors have been extensively studied as significant environmental risk factors associated with colon cancer, contributing to approximately 80% of cases. Moreover, factors such as Western diets, obesity, and high-fat intake have been shown to increase the risk of developing colon cancer and its recurrence [7,8]. Therefore, regulating lipid metabolism could be essential for controlling the progression of CRC.

Zinc-α2 glycoprotein (ZAG) is a 43 kDa polypeptide and has been classified as a novel adipokine due to its role in lipid mobilization [9]. ZAG has both lipolytic and lipogenic effects, and it promotes fat metabolism by interacting with β3 adrenergic receptors [10]. Recent research has demonstrated that ZAG treatment may enhance lipolysis in isolated adipocytes in both *in vivo* and *in vitro* studies involving humans and animals. In addition, ZAG can also regulate the metabolism of excess free fatty acids (FFA) produced by adipocytes during increased lipolysis [11,12]. Moreover, ZAG is recognized as a tumor marker, as it is overexpressed in various cancer types and is associated with weight loss when its levels are elevated. In both humans and rodents, ZAG expression in obesity is inversely correlated with body weight and fat mass [13]. For example, ZAG degradation promotes cholangiocarcinoma growth by inhibiting apoptosis through the tripartite motif-containing protein (TRIM) 25 [14]. Additionally, in triple-negative breast cancer, the release of ZAG promotes fibrosis within the tumor microenvironment [15]. ZAG is responsive to androgens and participates in AR-induced proliferation and metastasis of prostate cancer cells [16]. ZAG is a tumor suppressor in pancreatic cancer that induces mesenchymal–epithelial cell transdifferentiation by inhibiting TGF-β-mediated ERK signaling [17]. However, the role of ZAG in colorectal tumors has been inadequately explored, and the underlying mechanisms remain unclear.

This investigation aims to investigate the function of ZAG in CRC by knocking down the ZAG gene in CRC cells to assess its functional relevance and determine how such knockdown affects associated signaling pathways. Overall, our findings suggest that ZAG plays a significant role in the progression of CRC.

## 2 Methods

### 2.1 Cell lines and cell culture

Human CRC cell lines SW480, HCT116, and the intestinal epithelial cell line NCM460 were purchased from the Chinese Academy of Sciences Cell Bank and cultured in Dulbecco’s modified Eagle’s medium (DMEM) (Cytiva, Marlborough, USA) supplemented with 10% fetal calf serum (Gibco, Invitrogen, USA) and 1% penicillin/streptomycin (Gibco, Invitrogen, USA). All cells were maintained at 37°C in a 5% CO_2_ atmosphere.

### 2.2 Cell transfection

To facilitate gene knockdown, a ZAG plasmid was constructed, and a control shRNA plasmid was used as the negative control. The design, synthesis, and collection of all plasmids were performed by Suzhou Jima Pharmaceutical Co., Ltd. Lipofectamine 2000 (lot number: 2398587; Invitrogen) was utilized for transient transfections according to the manufacturer’s recommendations. A negative control (shNC) was used for experiments, and Western blotting was performed to verify the efficiency of ZAG knockdown.

### 2.3 Cell proliferation assay and colony formation assay

Cell growth was assessed using the counting kit-8 (CCK-8; Beyotime, China). Briefly, a total of 1,000 cells per well were seeded in a 96-well plate. After 72 h of incubation, the culture media was discarded, and the CCK-8 reagent was added to each well. After 1 h of incubation, cell proliferation was measured using a microplate reader (Bio-Tek).

For the colony formation assay, 500 cells per well were seeded in a 6-cm plate and cultured for 14 days in a medium containing 10% fetal bovine serum. After removing the growth media, the cells were fixed with methanol for 10 min, stained with crystal violet, washed with water, dried, and counted.

### 2.4 Apoptosis assay

The apoptotic rate of the cells was determined using the Annexin V-fluorescein isothiocyanate/propidium iodide kit (Beckman Coulter, Brea, CA). After staining, apoptosis rates were assessed and quantified by flow cytometry.

### 2.5 Oil red O staining

Coverslip-grown CRC cells were fixed for 15 min in 4% paraformaldehyde and subsequently stained for 30 min with a 3 mg/mL Oil Red O solution (Shanghai Yuanye Biological Co., Ltd.). Lipid droplets were visualized using a microscope (Olympus, DP73, Tokyo, Japan) and analyzed using ImageJ software after counterstaining with hematoxylin.

### 2.6 Western blot

The proteins were lysed on ice for 30 min using IP cell lysis buffer (Beyotime). The concentration of the extracted proteins was quantified using the Bicinchoninic Acid (BCA) protein assay kit (Thermo Fisher Scientific, Inc.). Then, the proteins were separated by electrophoresis on a 12% sodium dodecyl sulfate–polyacrylamide gel and transferred to a 0.45 µm polyvinylidene fluoride membrane. The membrane was blocked for 2 h using 1% bovine serum albumin to prevent non-specific binding. After blocking, the membrane was incubated overnight at 4°C on a shaker with diluted primary antibodies. Following this, the membrane was washed and incubated for 1 h with goat anti-rabbit IgG (1:2,000, ab7090; Abcam). The membrane was washed again before detection using the enhanced chemiluminescence substrate solution from Lulong Biotech.

The primary antibodies used were as follows: ZAG (1:1,000, ab180574, Abcam, UK), epithelial (E)-cadherin (1:1,000, ab40772, Abcam, UK), neural (N)-cadherin (1:1,000, ab76011, Abcam, UK), alpha-smooth muscle actin (α-SMA, 1:1,000, ab314895, Abcam, UK), acetyl-CoA carboxylase 1 (ACC1, 1:1,000, ab109368, Abcam, UK), ACC2 (1:1,000, ab287160, Abcam, UK), fatty acid synthase (FAS, 1:1,000, ab133619, Abcam, UK), phosphoinositide 3-kinase (PI3K, 1:1,000, ab267787, Abcam, UK), protein kinase B (AKT, 1:1,000, ab283852, Abcam, UK), phosphorylated AKT (p-AKT, 1:1,000, ab8805, Abcam, UK), mechanistic target of rapamycin (mTOR, 1:1,000, ab134903, Abcam, UK), phosphorylated mTOR (p-mTOR, 1:1,000, ab137133, Abcam, UK), and glyceraldehyde 3-phosphate dehydrogenase (1:1,000, ab8245, Abcam, UK).

### 2.7 Statistical analysis

The data were analyzed using GraphPad Prism 8 (GraphPad Software Inc., San Diego, CA) and are presented as mean ± standard deviation (SD). Statistical comparisons were performed using Student’s *t*-test or one-way analysis of variance, as appropriate, and *p* < 0.05 was considered statistically significant.

## 3 Results

### 3.1 ZAG is highly expressed in CRC

**Figure 1:**
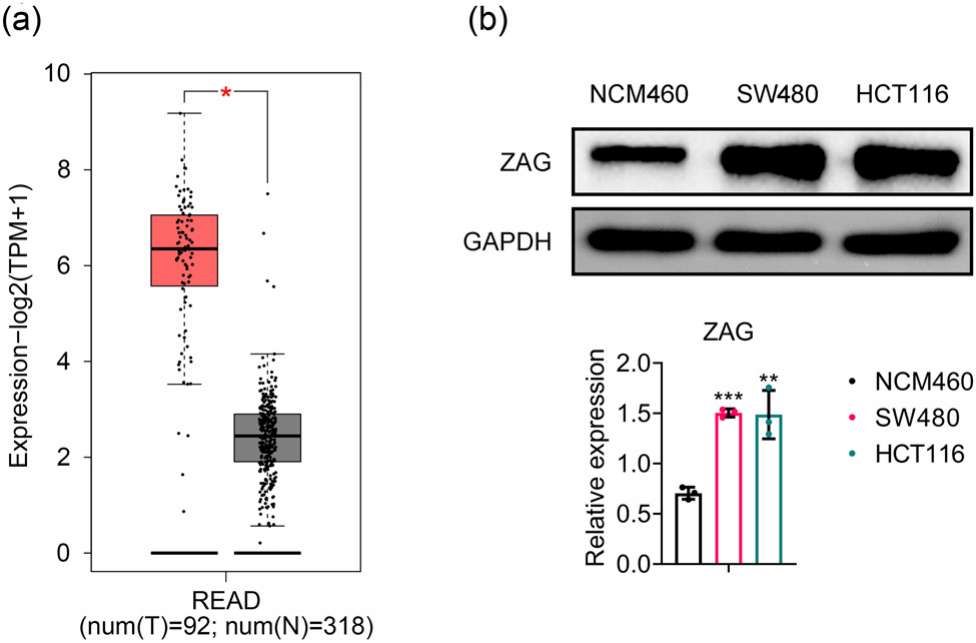
ZAG is highly expressed in CRC. (a) Analysis of ZAG mRNA expression in human rectal adenocarcinoma tissues compared to normal tissues using the GEPIA database. (b) Western blot analysis was performed to detect ZAG protein expression in the intestinal epithelial cell line NCM460 and CRC cell lines SW480 and HCT116. Values are presented as mean ± SD. **p < 0.01, ***p < 0.001 versus NCM460 group. *n* = 3.

The expression of ZAG was first investigated in rectal adenocarcinoma tissues and normal intestinal tissues using the GEPIA database, and we observed that rectal tumor tissues exhibited significantly higher levels of ZAG expression than normal intestinal tissues (Figure 1a). Next, Western blot analysis was performed on the intestinal epithelial cell line NCM460 and CRC cell lines SW480 and HCT116 (Figure 1b), and the results demonstrated a significantly elevated expression level of ZAG protein in CRC cells compared to the intestinal epithelial cell line. Collectively, these results suggest that ZAG expression is upregulated in CRC.

### 3.2 Knockdown of ZAG inhibits the growth of CRC cells

**Figure 2:**
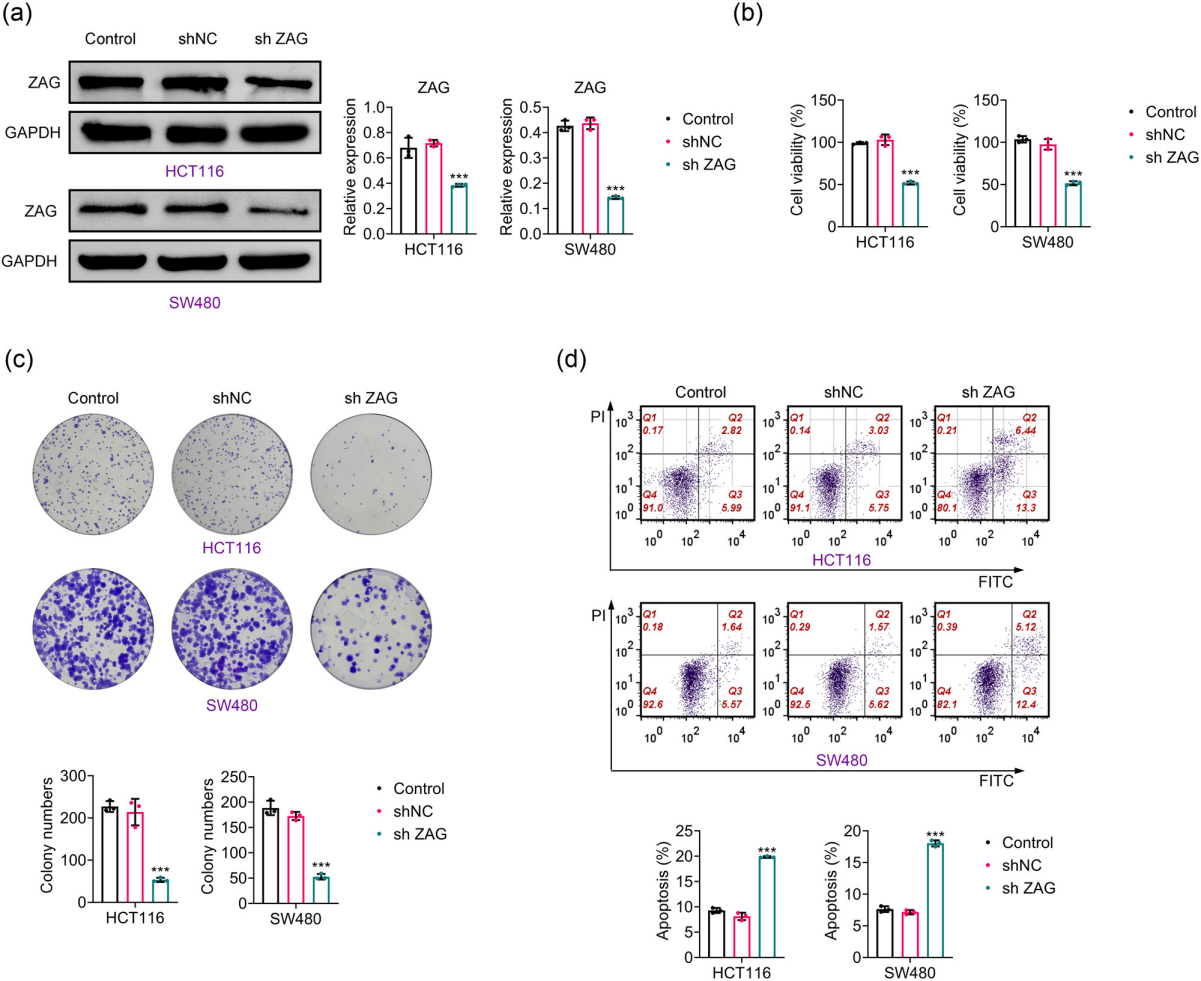
The knockdown of ZAG inhibits CRC cell growth. (a) Western blot analysis of ZAG expression in CRC cells following ZAG knockdown. (b) CCK-8 assay was conducted to measure cell viability. (c) Crystal violet staining was used to assess colony formation. (d) Flow cytometry was performed to determine the apoptosis rate of CRC cells. Values are presented as mean ± SD. ***p < 0.001 versus shNC group. *n* = 3.

To assess the role of ZAG, CRC cells were transfected with the ZAG shRNA plasmid to induce ZAG knockdown. Western blotting confirmed that ZAG shRNA significantly reduced ZAG expression in CRC cells, validating the successful creation of the shRNA plasmid (Figure 2a). The CCK-8 assay results demonstrated that ZAG knockdown inhibited the growth of CRC cells (Figure 2b). Furthermore, ZAG depletion was found to reduce colony formation in CRC cells (Figure 2c), and flow cytometry analysis revealed that ZAG knockdown significantly increased the apoptosis rate of CRC cells (Figure 2d). Overall, ZAG inhibition resulted in decreased survival of CRC cells.

### 3.3 Knockdown of ZAG inhibits EMT of CRC cells

**Figure 3:**
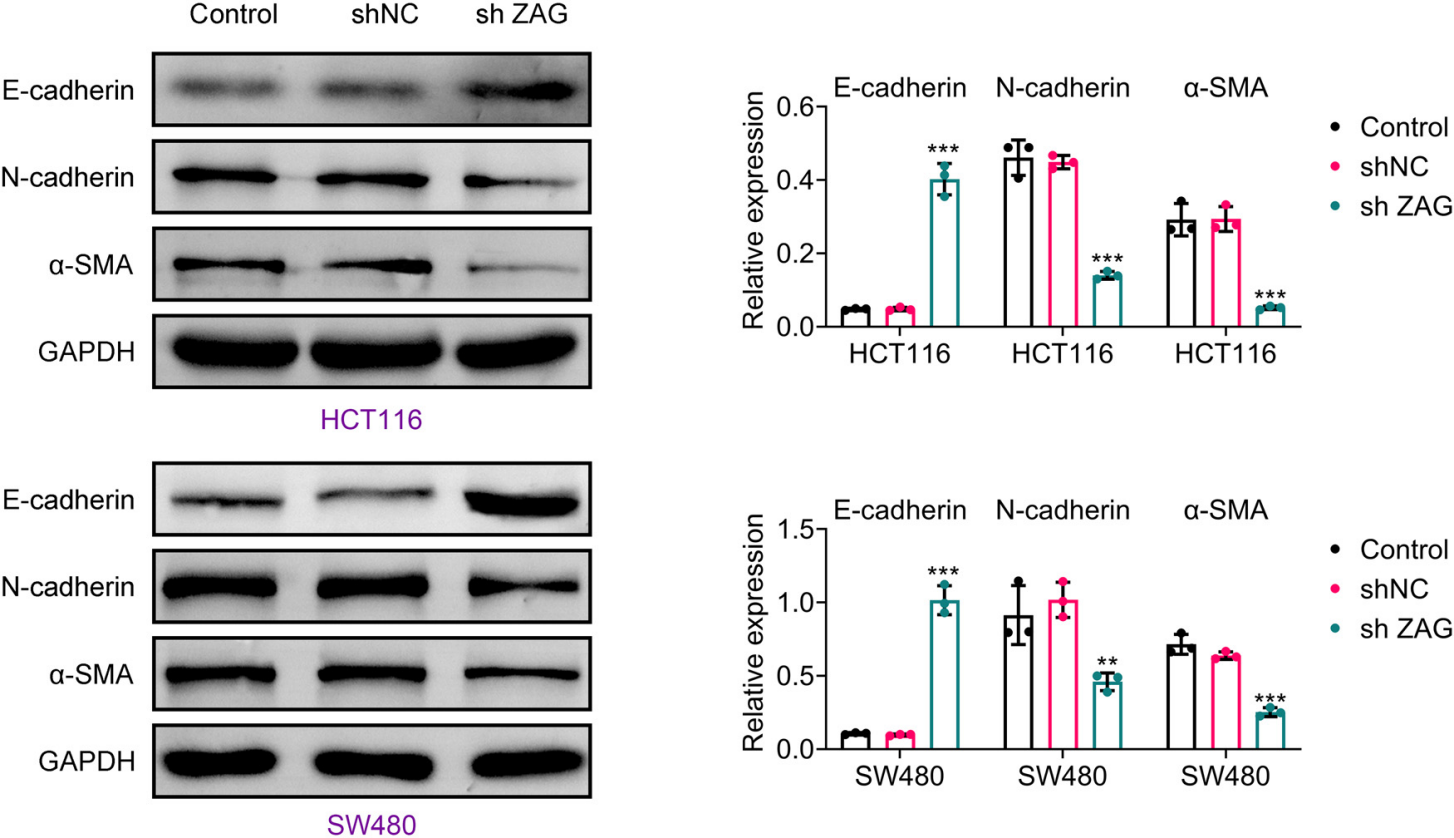
Knockdown of ZAG inhibits EMT of CRC cells. Western blot analysis was performed to detect the expression of E-cadherin, N-cadherin, and α-SMA proteins. Values are presented as mean ± SD. **p < 0.01, ***p < 0.001 versus shNC group. *n* = 3.

Western blot analysis was performed to evaluate the impact of ZAG on the epithelial–mesenchymal transition (EMT) in CRC cells, and we found that shZAG reduced the levels of EMT-associated proteins, including N-cadherin and α-SMA. Conversely, shZAG increased the expression of E-cadherin in CRC cells (Figure 3). Therefore, lowering ZAG levels can effectively inhibit EMT in CRC cells.

### 3.4 Knockdown of ZAG inhibits lipid synthesis in CRC cells

**Figure 4:**
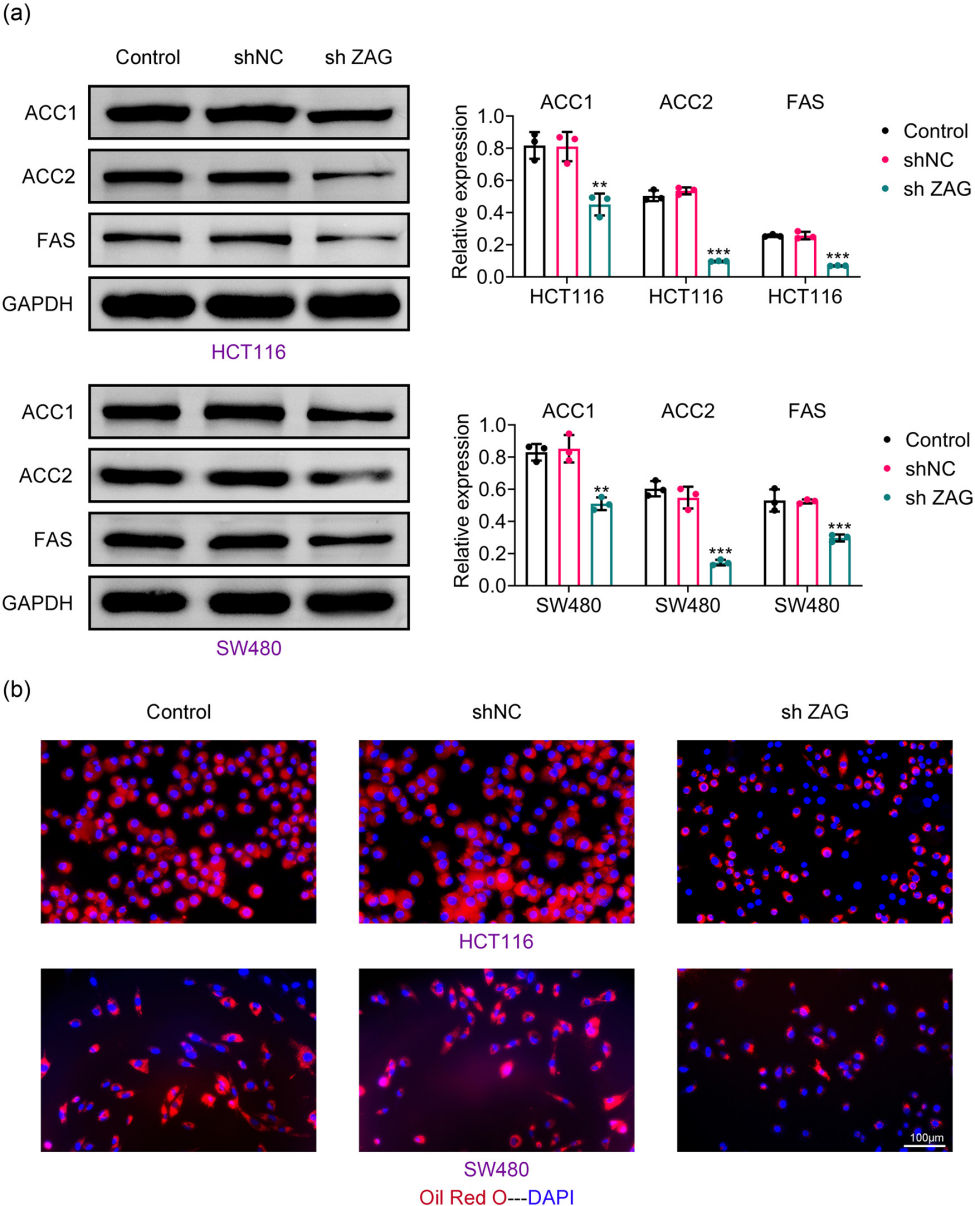
Knockdown of ZAG inhibits lipid synthesis in CRC cells. (a) Western blot analysis was conducted to detect the expression of ACC1, ACC2, p-ACC, and FAS proteins. (b) Oil Red O staining was used to quantify lipid droplet area. Values are presented as mean ± SD. **p < 0.01, ***p < 0.001 versus shNC group. *n* = 3.

To explore the relationship between ZAG and lipid production in CRC, we utilized protein immunoblotting and Oil Red O staining. Western blot analysis revealed a significant reduction in the levels of lipogenesis-related proteins FAS, ACC2, and ACC1 following ZAG knockdown (Figure 4a). Notably, ZAG is known to regulate adipogenesis. The results of Oil Red O staining indicated that the downregulation of ZAG was accompanied by a reduction in lipid droplet formation (Figure 4b). Thus, these findings suggest that ZAG inhibition can decrease lipid synthesis in CRC cells.

### 3.5 Knockdown of ZAG inhibits PI3K/AKT/mTOR signaling pathway

**Figure 5:**
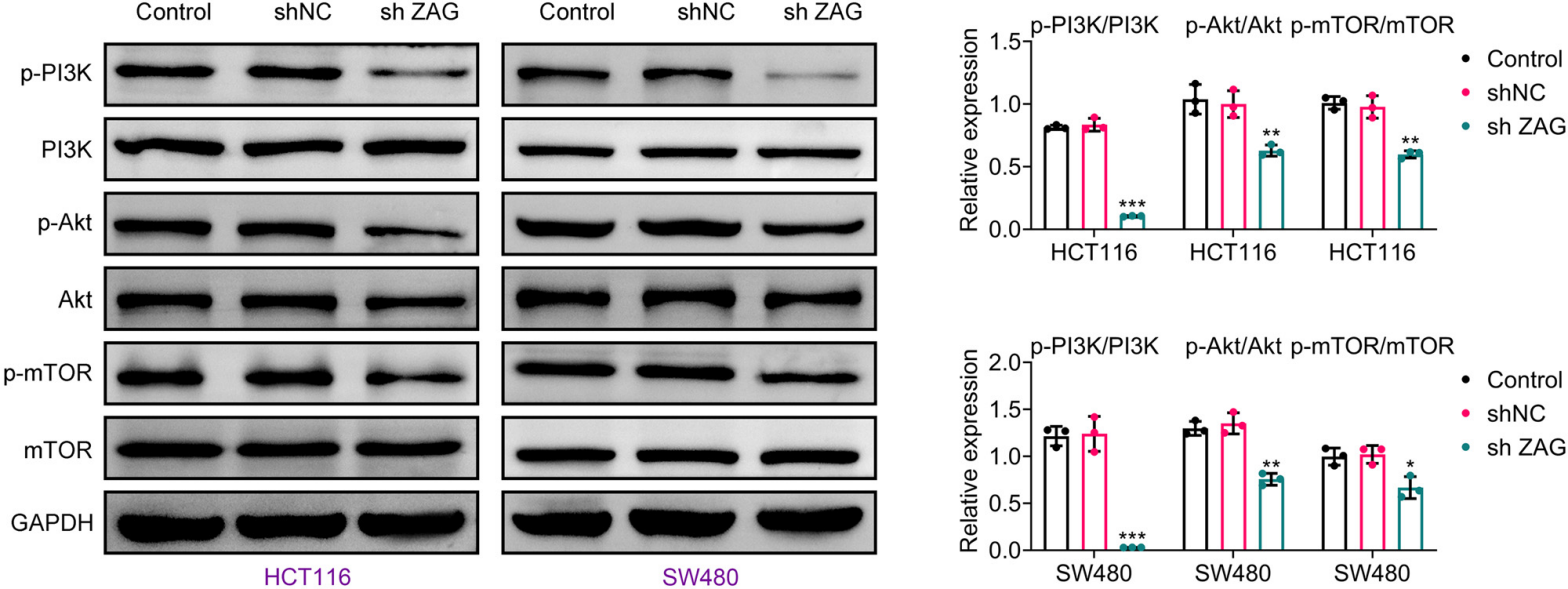
Knockdown of ZAG inhibits PI3K/AKT/mTOR signaling pathway. Western blot analysis was performed to detect the expression of PI3K, p-PI3K, AKT, p-AKT, mTOR, and p-mTOR proteins. Values are presented as mean ± SD. *p < 0.05, **p < 0.01, ***p < 0.001 versus shNC group. *n* = 3.

To further understand the molecular mechanisms underlying ZAG’s effects, we investigated its role in activating the PI3K/AKT/mTOR signaling pathway. Western blotting demonstrated that ZAG knockdown resulted in decreased expression levels of p-PI3K, p-AKT, and p-mTOR in CRC cells (Figure 5). This indicates that ZAG may promote cancer progression through the PI3K/AKT/mTOR signaling pathway.

## 4 Discussion

The formation of cancer is a complex process influenced by multiple factors, including genetics, nutrition, lifestyle choices, and genetic alterations [18], and identifying the genes that promote the onset and progression of CRC could significantly enhance its diagnosis and management. ZAG is predominantly secreted by mature adipocytes in healthy individuals, where it stimulates lipid mobilization in an autocrine or paracrine manner. Previous research has also indicated that malignant tumor cells express ZAG [19]. However, the specific impact of ZAG on CRC remains unclear. In this study, we demonstrated that ZAG is overexpressed in CRC tissues. Conversely, low ZAG expression was found to promote apoptosis and inhibit CRC cell proliferation. Additionally, ZAG inhibition suppressed the PI3K/AKT/mTOR signaling pathway, lipid production, and EMT. Our findings suggest that ZAG plays a crucial role in regulating lipid accumulation and colorectal carcinogenesis, indicating that targeting this protein could represent a viable therapeutic approach for CRC.

EMT is considered essential for tumor cells to acquire invasive and motile characteristics, facilitating metastasis and recurrence in various malignancies [20]. Cancer stem cells (CSCs) possess numerous protective mechanisms that enable them to withstand chemotherapy, making them a central focus in the study of aggressive cancers that currently lack effective treatments. In addition, numerous studies have shown that EMT is important for the enrichment of CSCs and their associated resistance to therapy [21,22]. Therefore, there is an emerging emphasis on understanding the biochemical components of CSCs and EMT in cancer therapy. E-cadherin, N-cadherin, and α-SMA are established markers of EMT [23]. In this study, our analysis indicates that low levels of ZAG expression effectively inhibit EMT.

Aberrant lipid metabolism is one of the earliest anomalies observed in tumor cells. Lipid synthesis not only produces a significant quantity of membrane phospholipids essential for tumor cell division and proliferation but also generates various lipid signaling molecules that promote cancer development [24]. Rapidly growing tumor cells have an increased demand for macromolecules, particularly lipids. When circulating lipids are insufficient to meet this demand, cancer cells compensate by enhancing de novo lipogenesis [25]. Given that CRC is reliant on lipid production, targeting lipid anabolism presents a potentially effective therapeutic strategy, and effective anti-CRC therapies likely depend on the inhibition of key molecules involved in lipid production [26]. Our results demonstrate that low ZAG expression inhibits lipid synthesis and accumulation, further supporting its potential as a therapeutic target in CRC.

Recent studies increasingly highlight the role of the PI3K/AKT/mTOR pathway in regulating various cellular processes, including adhesion, migration, survival, and proliferation [27]. The activation of AKT signaling promotes cell growth and tumor progression by regulating downstream cell cycle components [28]. In this pathway, phosphatidylinositol 4,5-bisphosphate (PIP2) is converted into phosphatidylinositol 3,4,5-trisphosphate (PIP3) by the lipid kinase PI3K. mTOR, a key protein in the PI3K/AKT/mTOR pathway, requires PIP3 as a second messenger for AKT translocation to the plasma membrane [29]. Furthermore, this pathway is crucial for lipid production and EMT [30].

The PI3K/AKT/mTOR pathway regulates the absorption and release of various amino acids and glucose in colon epithelial cells, influencing their responses to complex extracellular signals. This signaling pathway also plays a significant role in multiple cellular processes, transforming external stimuli into intracellular signals that affect metabolism and nutrient absorption. Thus, it has a profound impact on the onset, progression, metastasis, and prognosis of CRC [31,32]. The PI3K/AKT/mTOR pathway can also be activated by numerous growth factors, including vascular endothelial growth factor (VEGF), insulin-like growth factor 1 (IGF1), hormones, nutrients, and oxygen [33]. Herein, our study confirmed that the knockdown of ZAG can inhibit the expression of the PI3K/AKT/mTOR pathway in CRC cells.

## 5 Conclusion

In conclusion, the deletion of ZAG can effectively block the PI3K/AKT/mTOR signaling pathway, thereby preventing tumor cell proliferation, EMT, and lipid production in CRC. Our findings indicate that ZAG is an oncogene in CRC and a viable therapeutic target, particularly for patients with aberrant activity in the PI3K/AKT/mTOR pathway. However, due to the absence of *in vivo* and clinical investigations, further investigations are required to confirm these conclusions.
